# Prevalence of under-nutrition and associated factors among patients with liver cirrhosis at a tertiary hospital in Ethiopia

**DOI:** 10.1097/MD.0000000000041226

**Published:** 2025-01-03

**Authors:** Biruk Mulugeta, Henok Fisseha, Abel Mureja Argaw, Rodas Kassu, Hailemichael Desalegn

**Affiliations:** a Department of Internal Medicine, St. Paul’s Hospital Millennium Medical College, Addis Ababa, Ethiopia; b Division of Neurosurgery, University of Wisconsin, IL.

**Keywords:** associated factors, ethiopia, liver cirrhosis, liver disease, nutrition, under-nutrition

## Abstract

Liver cirrhosis is a major health burden, resulting in over 1 million deaths per year worldwide. Nutritional imbalance often complicates the course of liver diseases, particularly of cirrhosis and has been linked to increased mortality. Despite the high disease burden, there is paucity of literature regarding the magnitude of under-nutrition in patients with cirrhosis and its associated factors in Ethiopia and sub-Saharan Africa. The study aimed to assess the prevalence of under-nutrition and its associated factors among adult out-patients with liver cirrhosis. A hospital-based cross-sectional study was conducted among 136 adult out-patients with cirrhosis who visited the hepatology clinic of St. Paul’s Hospital Millennium Medical College. Data were obtained through patient interviews, medical record reviews, anthropometric and handgrip strength measurements and collected using a structured checklist and analyzed using Statistical Package for the Social Sciences (SPSS) version 26.0. Body mass index with cutoff points adjusted for the degree of ascites were used to diagnose under-nutrition. Descriptive statistical tools and binary and multivariable logistic regression analyses were employed, and statistical significance was set at <0.05. The mean age of study participants was 39.5 years (standard deviation: ±11.2) and 62.5% were males. Chronic hepatitis B virus infection (57.4%) was the most common cause of liver cirrhosis, followed by alcohol-associated liver cirrhosis (12.5%). The majority (70.6%) of the study participants were undernourished. The factors found to have statistically significant association with under-nutrition were, rural area of residence (adjusted odds ratios [AOR]: 5.65, 95% confidence interval [CI]: 1.98–16.1), presence of ascites (AOR: 2.43, 95% CI: 1.03–5.7) and the disease severity, as measured by the child–pugh class (AOR, 1.11; 95% CI: 0.45–2.7). Under-nutrition was found to be a common problem among out-patients with liver cirrhosis and patients from rural areas and those with advanced disease were disproportionately affected. It is imperative to implement routine nutritional screening and plan on appropriate interventions for patients with liver cirrhosis.

## 1. Introduction

Liver cirrhosis is the final and irreversible stage of chronic liver disease, which is characterized by a process of continuous inflammation, destruction and regeneration of liver parenchyma.^[1]^ Worldwide, it is a major cause of mortality accounting for over a million deaths per year.^[2]^ Sub-Saharan Africa has the second highest age-standardized death rate caused by cirrhosis, with chronic viral hepatitis as the leading cause.^[2,3]^ Despite some regional variations, the major causes of chronic liver disease leading to cirrhosis are chronic viral hepatitis (hepatitis B virus and hepatitis C virus), alcohol-associated liver disease and nonalcoholic fatty liver disease.^[3]^ Patho-physiologically, cirrhosis is a progressive disease, which runs through a fairly stable initial course termed compensated stage, followed by the decompensated stage which is marred by multitude of complications including nutritional imbalances.^[3,4]^ Since the liver is a vital organ that is responsible for an array of metabolic functions that help support body’s nutritional balance, the progressive loss of liver function due to cirrhosis poses serious threat to patient’s nutritional state.^[4,5]^ Several factors, such as the cause and stage of cirrhosis, degree of ascites, and presence of other comorbidities influence nutritional status.^[5,6]^ Besides the loss of liver's metabolic function, other factors also contribute to the poor nutritional state of cirrhotic patients, including, poor intake, altered gut motility and absorption, heightened basal metabolic rate and physician-led dietary protein restriction.^[6,7]^ The course and outcome of liver cirrhosis is much worse when compounded by under-nutrition, as studies show it to be an independent predictor of disease progression and mortality.^[7–9]^

Furthermore, under-nutrition in cirrhosis is a concern as it mainly results from the loss of skeletal muscle mass (sarcopenia), which by its own stand is associated with a worse quality of life, high rates of complications, and increased mortality.^[8,9]^

According to several studies, disease stage appears to be the single most important factor to influence the magnitude of under-nutrition present, to this end; most studies have reported prevalence rates of 50% to 90% in the advanced stages of cirrhosis.^[10–13]^ The high prevalence rates has pushed professional societies to recommend a routine nutritional screening for patients with cirrhosis, however, the process of assessing under-nutrition in cirrhosis is complex because most nutritional markers used conventionally are proteins produced in the liver, and the presence of ascites and peripheral edema limits the use of commonly used anthropometric measurements.^[14,15]^

Among the anthropometric measures, body mass index (BMI) is the easiest and least costly; however, as it heavily relies on body weight measurement, its use in volume overloaded states has been challenged.^[15,16]^ To address this particular drawback, higher BMI cutoff points that take into account the degree of ascites and peripheral edema have been validated.^[17]^ Other measures that are less affected by body fluid composition include the mid-arm muscle circumference and triceps skin-fold test but require trained personnel for better inter- and intra-observer agreement, making them less suitable in busy out-patient clinics.^[17]^

Furthermore, as busy clinicians continue to search for methods that are simple, reliable and cost effective, functional measurements such as handgrip strength (HG) are becoming more popular.^[18]^ HG is measured in kilogram force using a portable hydraulic dynamometer adjusted to patient hand size and multiple studies support HG to be a highly sensitive indicator of functional impairment, reflective of protein-calorie malnutrition, and correlates well with hard clinical outcomes.^[19–21]^

In conclusion, literature regarding the significance of under-nutrition among this group of patients is increasing; however, most studies are restricted to middle- and high-income countries, and little is known about the prevalence of under-nutrition among patients with cirrhosis in low income countries.

In Ethiopia, there are few studies exploring liver cirrhosis and its etiology, and the available data indicate that chronic liver disease is a common reason for hospital admissions, with the majority being due to viral hepatitis.^[22,23]^ Furthermore, it is worth noting that studies regarding under-nutrition are still notably absent in the context of Ethiopia, therefore, this study aimed to determine the prevalence of under-nutrition and its associated factors among patients with liver cirrhosis at St. Paul’s Hospital Millennium Medical College (SPHMMC) Hepatology out-patient clinic.

## 2. Materials and methods

### 2.1. Study design, setting, and population

This hospital-based, cross-sectional study was conducted from May 18th to October 16th 2020, at hepatology clinic of SPHMMC, Addis Ababa, Ethiopia, one of the largest government-owned tertiary and teaching hospitals, providing services for patients referred from all corners of the country.^[24]^

The sample size was calculated using a single-population proportion formula. The value of P was taken 50% as no study exploring the prevalence of under-nutrition among patients with liver cirrhosis in Ethiopia was obtained. Desired confidence level of 95% and margin of error of 5.5% were used. This led to a sample of (n_o_ = 318).

Patient flow trend during the same period of the preceding year (May 1st–October 30th 2019) was used to estimate patient flow which was <10,000. Therefore, the calculated sample size (n_o_ = 318) was adjusted using the Slovin equation, sample size calculation for a small population, and resulted in a sample size of (n = 135).

Consecutive patients aged ≥18 years, diagnosed with liver cirrhosis, and who visited the out-patient clinic during the study period were included. Liver cirrhosis was defined as the presence of cirrhosis based on a combination of clinical, laboratory, and radiologic findings in the patient’s medical records.

To avoid confounding effects on nutritional status, patients co-diagnosed with malignancies, including hepatocellular carcinoma, inflammatory bowel disease, or chronic diarrhea were excluded from the study. Likewise, patients with any physical limitation that compromised the anthropometric assessments were excluded.

### 2.2. Operational definitions

The following operational definitions were applied:

Severity of ascites: the international ascites club^(R)^ definitions were used, as follows^[25]^:

- Mild ascites (grade 1): no clinically evident ascites only detectable by ultrasound.
- Moderate ascites (grade 2): moderate abdominal distention.
- Large/tense ascites (grade 3): marked abdominal distention.

Under-nutrition in patients with cirrhosis: it is defined using BMI cut-off values modified for the degree of fluid retention (ascites), as validated and suggested in a previous study^[17]^; cut-off values were obtained and structured into table format (Table 1).

**Table 1:** Modified body mass index cut-off points to define malnutrition in patients with cirrhosis.

Nutritional status	Body mass index cut-off points modified for degree of ascites		
	Mild ascites	Moderate ascites	Severe ascites
Preserved	≥22	≥23	≥23
Mild malnutrition	<22	<23	<25
Moderate malnutrition	<19	<20	<22
Severe malnutrition	<16	<17	<18

### 2.3. Data collection procedures and quality management

Data were collected using a structured data collection checklist that was prepared by reviewing different literatures. Due to coronavirus disease 2019 outbreak and subsequent decrease in out-patient clinic visits, pilot stage was limited to 10 patients with liver cirrhosis in the SPHMMC out-patient clinic. The data collection process consisted of 3 parts: a structured interview; measuring weight, height, handgrip strength, and severity of ascites; and reviewing patient medical records. Data regarding dietary diversity were collected using the Food and Agriculture Organization Guidelines for measuring household and individual dietary diversity by 24 hours recall method.^[26]^ To minimize intra-observer variability, one trained general practitioner performed all anthropometric measurements. Body weight in kilograms and height in centimeters were measured using a digital scale and stadiometer. Handgrip strength was measured using a mechanical handgrip dynamometer (CAMRY EH101), using the non-dominant hand. Three readings were noted with a gap of more than 30 seconds, and the mean of the 3 recordings was taken for further analysis. Normal reference values for handgrip were obtained according to age- and sex-matched data.^[27]^ All values were recorded in kilograms.

### 2.4. Data processing and analysis

Data were analyzed using Statistical Package for the Social Sciences version 26.0 (SPSS Inc., Chicago, IL). Descriptive statistics, including mean, median, standard deviations, and percentages, were calculated, and logistic regression was used to examine the association between under-nutrition and associated factors. Variables with a *P* value < .2 in the univariable analysis were included in the multivariable logistic regression model. All variables in the multivariable model were considered statistically significant at *P* < .05. Associations between variables are presented as adjusted odds ratios (AOR) with 95% confidence interval (CI).

### 2.5. Ethical consideration

The study was conducted in accordance with the guidelines of the Declaration of Helsinki and was approved by the Institutional Review Board of St. Paul’s Hospital Millennium Medical College with reference number P.M.23/740. To ensure patient autonomy, informed written consent was obtained from each participant and data were obtained using an anonymous questionnaire.

## 3. Results

### 3.1. Socio-demographic characters

The study included 136 patients with liver cirrhosis; males represented the majority 85 (62.5%). Patients’ age was widely distributed; ranging from 18 to 85 years with a mean age of 39.5 years (standard deviation [SD]: ±11.2). Most participants 93 (68.4%) were married and majority 111 (81.6%) reported to have attended formal education. Large numbers of participants were from urban areas 84 (61.8%) (Table 2).

**Table 2:** Participant’s socio-demographic characteristics (n = 136), SPHMMC, Addis Ababa, Ethiopia, 2020.

Characteristics	Number	Percent (%)
Age group		
18–29	29	21.3
30–49	77	56.6
≥50	30	22.1
Gender		
Male	85	62.5
Female	51	37.5
Marital status		
Married	93	68.4
Single	31	22.8
Divorced/widowed	12	8.8
Level of education		
Primary	42	30.9
Secondary	41	30.1
College and above	28	20.6
Can’t read and write	18	13.2
Can read and write	7	5.1
Occupation		
Farmer	19	14.0
Government employee	21	15.4
Merchant	7	5.1
Others (self-employed, daily labor)	63	46.3
Unemployed	25	18.4
Monthly income (Ethiopian Birr)		
<500	27	19.9
500–1499	46	33.8
1500–2999	25	18.4
3000–4499	21	15.4
5000–10,000	17	12.5
Area of residence		
Urban	84	61.8
Rural	52	38.2

SPHMMC = St. Paul’s Hospital Millennium Medical College.

### 3.2. Dietary diversity

Patients reported to have mostly consumed carbohydrates (71%–85%), dark green and vitamin A rich vegetables (80%), or other vegetables like onions, carrots, and tomatoes (94%) in the preceding 24 hours.

On the contrary, consumption of foods containing protein and fat was grossly inadequate; 29.4% of patients consumed flesh of any kind, 26.5% of patients consumed poultry and dairy products, and only 24 (17.6%) patients consumed foods containing fats, like oil or butter (Table 3).

**Table 3:** Nutritional diversity (n = 136), SPHMMC, Addis Ababa, Ethiopia, 2020.

Food group	Number (n = 136)	Percent
Any cereals (bread, rice noodles, biscuits, or any other foods made from millet, sorghum, maize, rice, and wheat)	116	85
Any potatoes	97	71
Dark green leafy and vitamin A rich vegetables	109	80
Other vegetable’s (carrot, tomato, and onion)	128	94
Vitamin A rich fruits	77	56.6
Organ meats (liver, kidney, and heart)	7	5.1
Flesh (meat) any beef of, lamb, goat, sheep, and chicken	40	29.4
Any eggs	36	26.5
Legumes, nuts and seeds (any foods made from beans, peas, lentils, and nuts)	87	64
Dairy products	40	26.4
Any foods made with oil, fat, or butter	24	17.6

SPHMMC = St. Paul’s Hospital Millennium Medical College.

### 3.3. Medical factors

The commonest cause of liver cirrhosis was chronic hepatitis B virus infection 78 (57.4%), followed by alcohol-associated liver cirrhosis 17 (12.5%) and chronic hepatitis C virus infection accounted for 13 (9.6%). More than two-thirds of the participants 107 (78.6%) were found to have at least moderate ascites. Regarding the overall disease severity, the majority 120 (88.2%) had decompensated cirrhosis, of which 86 (63.2%), 34 (25%), were child–pugh class B and C, respectively (Table 4).

**Table 4:** Summary of medical factors in liver cirrhosis patients (n = 136), SPHMMC, Addis Ababa, Ethiopia, 2020.

Medical factor	Number	Percent
Cause of cirrhosis		
Chronic HBV	78	57.4
Alcohol associated liver disease	17	12.5
Chronic HCV	13	9.6
Others	28	20.6
Degree of ascites		
Mild ascites	29	21.3
Moderate ascites	83	61.0
Severe (tense) ascites	24	17.6
Severity of cirrhosis		
CP class A	16	11.8
CP class B	86	63.2
CP class C	34	25.0

CP = child–pugh, HBV = hepatitis B virus, HCV = hepatitis C virus, SPHMMC = St. Paul’s Hospital Millennium Medical College.

### 3.4. Nutritional status of patients with cirrhosis

The minimum and maximum BMI values were 15.57 and 33.08 kg/m^2^, respectively with a mean BMI of 21.9 kg/m^2^ (SD ± 3.244). Compared to the optimal BMI cut-off points adjusted for patients with liver cirrhosis and ascites, which are, BMI of ≥22, ≥23, and ≥25 for patients with mild, moderate and tense ascites, respectively, most of the study participants 96 (70.6%) registered a value below the optimal BMI cutoff that defines preserved nutritional status. On further analysis, among the 96 undernourished patients, 55 (40.4%), 37 (27.2%), 4 (2.9%) had mild, moderate, and severe malnutrition, respectively.

### 3.5. Handgrip strength measurement

The mean HG strength of the patients was 25.1 kg (SD ± 8.4), with minimum and maximum values of 5.4 and 50.7 kg, respectively. Most of the participants 86 (63.2%) were found to have a weak hand grip strength compared to the age- and sex-matched reference values. On further analysis, patients grip strength was found to be related to the severity of under-nutrition, as HG was weak in all patients with severe, in (75%) of patients with moderate and in (56%) of patients with mild under-nutrition. Similarly, the grip strength was compared between patients with different degrees of cirrhosis severity, and 56%, 61%, and 70% of patients with child class A, B, and C had weak grip strength, respectively. Despite the above findings, handgrip strength was found to be weak in 23 (57.5%) of participants with preserved nutritional status.

### 3.6. Factors associated with under-nutrition

Patients from rural areas were found to have more than 5 times higher odds of being undernourished than their urban counterparts (AOR: 5.65, 95% CI: 1.98–16.1), however other socio-demographic factors did not show statistically significant association. Participants with moderate or higher degree of ascites were more than 2 times more likely to be undernourished than those with no ascites with (AOR: 2.43, 95% CI: 1.03–5.75).

Likewise, disease severity, as measured by the child–pugh class, showed a statistically significant association with nutritional status. To this end, compared with patients in child–pugh A, those in child–pugh C had slightly higher odds being undernourished (AOR: 1.11, 95% CI: 0.45–2.7). Other medical factors were not significantly associated with nutritional status (Table 5).

**Table 5:** Logistic regression analysis of associated factors of patients with liver cirrhosis (n = 136), SPHMMC, Addis Ababa, Ethiopia, 2020.

Variables	Significance (*P*-value)	COR (95% CI)	AOR (95%)
Gender	.698	0.861 (0.404–1.855)	–
Place of residence	<.001	6.71 (2.42–18.6)	5.65 (1.98–16.1)
Level of education	.059	8.05 (0.922–70)	–
Monthly income	.191	0.321 (0.86–1.98)	–
Serum albumin	.260	1.318 (0.815–2.131)	–
Ascites	.001	3.16 (1.609–6.195)	2.44 (1.03–5.75)
Child–pugh class	.018	1.11 (0.45–2.7)	1.07 (0.48–2.54)

AOR = adjusted odds ratio, COR = crude odds ratio, SPHMMC = St. Paul’s Hospital Millennium Medical College.

## 4. Discussion

According to this study, under-nutrition was found among 70.6% of the study participants. The place of residence, presence of ascites, and child–pugh class were found to be independently associated with the nutritional status of the patients.

The mean age of the participants was 39.51 years (SD ± 11.2), which is younger than previous studies; which reported mean age of participants 50.9 ± 11.1^[28]^ and 57.41 ± 10 years.^[29]^ A possible explanation could be in our study, the most common cause (67%) of liver cirrhosis was chronic viral hepatitis, which results in liver cirrhosis at young age compared to alcohol associated liver disease, the commonest etiology in the studies compared, which tends to occur late.

The results of this study showed the prevalence of under-nutrition among patients with liver cirrhosis to be (70.6%), although the number was alarmingly high; it is in agreement with most published studies which reported rates in the order of 65% to 90%.^[10–13]^ One possible explanation in this study can be the poor nutritional diversity reported by patients, as most patients heavily rely on a diet rich in carbohydrates but poor in terms of protein and fat content. Whether this is due to the inability to access or perceived fear of animal products worsening the liver disease requires further exploration. In our study, only 29.6% of patients had preserved nutritional status, echoing the findings from Brazil,^[28]^ Netherlands,^[30]^ and Tunisia.^[31]^ This finding confirms the important relationship between under-nutrition and liver cirrhosis, as has been observed in other studies.^[29–31]^

In the present study, ascites negatively affected the nutritional status of patients; 83.3% of patients with tense, 77.1% of patients with moderate ascites were undernourished compared with patients with no ascites (41.3%). Similar findings have been previously reported.^[29–32]^ A possible explanation for the high degree of under-nutrition in patients with tense ascites can be the gastric compression effect of ascites, with resultant early satiety, associated bowel edema, or ascites being an indicator of liver disease severity. This requires more studies with appropriate design to completely understand the cause-and-effect relationship.

Likewise, the severity of under-nutrition increased as a function of the child–pugh disease class. A similar pattern was reported by most studies,^[28–31]^ in contrast with an earlier study.^[33]^ One possible explanation for the deterioration of nutritional status in patients with decompensated cirrhosis (child–pugh B and C) can be the stringent dietary restrictions given to patients by health care providers, disease-related anorexia, and a persistent hypermetabolic state.^[28]^

In our study, the commonest cause of liver cirrhosis was chronic viral hepatitis (67%), but the cause of cirrhosis was not shown to have statistically significant association with nutritional status. This finding is similar to that of studies from France,^[17]^ Brazil,^[28]^ and the Netherlands.^[30]^ This may be due to the convergence in the mechanisms of under-nutrition, irrespective of the underlying cause. One study^[30]^ has reported that under-nutrition in liver cirrhosis is more common in the elderly, but our findings did not support this association.

One peculiar finding not reported in other studies is that patients with cirrhosis from rural areas were found to be disproportionately undernourished compared to their urban-dwelling counterparts. This difference may be the result of the poor availability of processed food items and supplements in rural areas, misunderstandings of recommended dietary advices, or other socio-cultural factors. Further studies are required to explore and confirm these results.

Regarding the assessment of handgrip strength, the findings of this study confirmed the association between weak HG and the severity of under-nutrition. This finding is comparable to those of previous studies.^[18,20,21]^ On the other hand, a significant number of patients 23 (57.5%) were found to have weak grip strength despite having preserved nutritional status, which might be due to the HG cutoff points being non-validated for the Ethiopian population, which needs further exploration with appropriate design.

As to our knowledge, this study is the first of its kind to evaluate the burden of under-nutrition and its associated factors in patients with cirrhosis in the study area. It employed a validated tool to assess the level of under-nutrition. This study is also the first to use a handgrip strength dynamometer to explore the relationship between grip strength, nutritional status and severity of cirrhosis in the Ethiopian context.

However, this study has several limitations. The cross-sectional study design prevented us from making definitive conclusions about associated factors. The reference values used for normal HG strength have not been validated in the Ethiopian population, which could have affected our findings.

## 5. Conclusions

This study shows more than two-thirds (70.6%) of patients with liver cirrhosis had at least some degree of under-nutrition. The factors found to have a significant association were place of residence, presence of ascites, and severity of liver disease, as measured by the child–pugh class. Physicians caring for patients with cirrhosis should be cognizant of the high burden of under-nutrition and plan on appropriate nutritional rehabilitation. Future well-controlled studies, that factor in the limitations of this study, should be encouraged.

## Acknowledgments

The authors wish to thank the staff of St. Paul’s Hospital Millennium Medical College and patients who participated in this study.

## Author contributions

**Conceptualization:** Biruk Mulugeta.

**Data curation:** Biruk Mulugeta, Henok Fisseha, Abel Mureja Argaw, Rodas Kassu, Hailemichael Desalegn.

**Formal analysis:** Biruk Mulugeta.

**Funding acquisition:** Biruk Mulugeta.

**Investigation:** Biruk Mulugeta, Henok Fisseha.

**Methodology:** Biruk Mulugeta.

**Project administration:** Biruk Mulugeta, Henok Fisseha, Abel Mureja Argaw, Rodas Kassu.

**Resources:** Biruk Mulugeta.

**Software:** Biruk Mulugeta.

**Supervision:** Biruk Mulugeta, Rodas Kassu, Hailemichael Desalegn.

**Validation:** Biruk Mulugeta.

**Visualization:** Biruk Mulugeta.

**Writing – original draft:** Biruk Mulugeta.

**Writing – review & editing:** Biruk Mulugeta, Henok Fisseha, Abel Mureja Argaw, Rodas Kassu, Hailemichael Desalegn.
